# Therapieansprechen und Komplikationen von älteren Patienten mit ANCA(antineutrophile cytoplasmatische Antikörper)-assoziierten Vaskulitiden

**DOI:** 10.1007/s00391-022-02145-0

**Published:** 2022-12-19

**Authors:** Lena Schulte-Kemna, David Kühne, Lukas Bettac, Hannes Herrmann, Ulla Ludwig, Martin Kächele, Bernd Schröppel

**Affiliations:** Klinik für Innere Medizin I – Sektion Nephrologie, Universitätsklinikum Ulm, Albert-Einstein-Allee 23, 89081 Ulm, Deutschland

**Keywords:** Gebrechlichkeit, Rheumatologie, Nierenerkrankung, Funktioneller Status, Mortalität, Frailty, Kidney disease, Rheumatology, Physical function, Survival

## Abstract

**Hintergrund:**

Von ANCA(antineutrophile cytoplasmatische Antikörper)-assoziierten Vaskulitiden (AAV) sind v. a. Menschen höheren Alters betroffen, ohne dass adaptierte Therapiekonzepte für diese Patienten existieren.

**Ziel der Studie:**

Ziel der Studie war es, Unterschiede in Verlauf und Outcome von Patienten mit AAV hinsichtlich des Alters zu analysieren.

**Material und Methoden:**

62 Patienten wurden auf Therapieansprechen, 53 (85 %) davon konnten hinsichtlich unerwünschter Nebenwirkungen (AE und SAE), analysiert werden. Es wurden ältere (> 65 J.) mit jüngeren (< 65 J.) Patienten verglichen. Das Therapieansprechen wurde nach 6 Monaten, Komplikationen wurden über 18 Monaten beurteilt.

**Ergebnisse:**

Das Therapieansprechen war in beiden Altersgruppen nicht unterschiedlich. In der multiplen logistischen Regression waren die pulmonale Beteiligung (OR = 6,9, KI = 1,7–27,8; *p* < 0,01) und die ΔGFR [ml/min] (OR = 0,93, KI = 0,89–0,97; *p* < 0,01) Prädiktoren für SAE. 14 Patienten hatten mehr als eine SAE; auch hier war eine pulmonale Manifestation bei Diagnose (28,2 % vs. 78,6 %, *p* < 0,01) Risikofaktor. Ältere Patienten (78,6 % vs. 43,6 %, *p* = 0,025) waren häufiger betroffen. Patienten mit mehreren SAE bekamen länger Glukokortikoiddosen über 5 mg/Tag (171 ± 65 Tage vs. 120 ± 70 Tage, *p* = 0,03).

**Diskussion:**

Hinsichtlich des Therapieansprechens wurden keine Unterschiede zwischen älteren und jüngeren Patienten gefunden. Bei älteren Patienten traten häufiger mehrere SAE auf. Es bestand eine Korrelation zwischen pulmonaler Manifestation und Dauer einer Glukokortikoidgabe mit einem komplizierten Verlauf. Die häufigsten SAE waren Infektionen, welche einer stationären Aufnahme bedurften.

**Schlussfolgerung:**

Die Therapie für ältere Patienten sollte individualisiert werden, mit dem Ziel einer raschen Reduktion von Glukokortikoiden. Ein besonderes Monitoring ist für ältere Patienten v. a. mit pulmonaler Manifestation bei Krankheitsbeginn angezeigt.

**Zusatzmaterial online:**

Zusätzliche Informationen sind in der Online-Version dieses Artikels (10.1007/s00391-022-02145-0) enthalten.

## Abstract (en)

**Background:**

ANCA (antineutrophil cytoplasmatic antibody)-associated vasculitis (AAV) mainly affects elderley people but adjusted therapy concepts for this patient group are lacking.

**Aim:**

The aim of this study was therefore to analyze differences in course and outcome of patients with AAV with respect to age.

**Materials and methods:**

62 patients were analyzed for treatment response, of whom 53 (85%) experienced adverse events (AE and SAE) that could be evaluated. Older (> 65 yrs.) versus younger (< 65 yrs.) patients were compared. Treatment response was assessed at 6 months, complications were assessed over 18 months.

**Results:**

Treatment response was not seen to differ by age groups. In multiple logistic regression, pulmonary involvement (OR = 6,9; CI = 1,7–27,8, *p* < 0,01) and ΔGFR [ml/min] (OR = 0,93; CI = 0,89-0,97, *p* < 0,01) were predictors of SAE. 14 patients had more than 1 SAE. Again, pulmonary involvement (28,2% vs. 78,6%, *p* < 0,01) was a risk factor and older patients (78,6% vs. 43,6%, *p* = 0,025) were more frequently affected. Patients with multiple SAEs received glucocorticoids of more than 5 mg/d for longer periods of time (171 ± 65 days vs. 120 ± 70 days, *p* = 0,03).

**Discussion:**

No differences were found between older and younger patients with regard to treatment response. Multiple SAEs occurred more frequently in elderly patients. There was a correlation between pulmonary manifestation and duration of glucocorticoid therapy with a complicated course. The most frequent SAEs were infections requiring hospitalisation.

**Conclusion:**

Therapy for elderly patients should be individualized with the goal of a fast reduction of glucocorticoids. Special monitoring is indicated for elderly patients, especially those with pulmonary involvement.

## Hintergrund und Fragestellung

ANCA(antineutrophile cytoplasmatische Antikörper)-assoziierte Vaskulitiden (AAV) können sich vielfältig präsentieren und betreffen – außer im Fall der renal limitierten Form – oft mehrere Organsysteme. Mit einer Inzidenz von ca. 20/10^6^ Einwohnern gehören AAV zu den seltenen Erkrankungen [3]. Das mediane Alter bei Diagnose liegt zwischen 60 und 65 Jahren [4], mit einem Inzidenzgipfel bei 70 bis 75 Jahren [5]. Viele der AAV-Interventionsstudien schließen ältere Patienten allerdings aus oder analysieren die Ergebnisse nicht altersabhängig [1, 2].

Unsere Hypothese ist, dass die leitliniengerechte Therapie der AAV bei älteren Patienten (> 65 Jahren) im Vergleich mit jüngeren Patienten (< 65 Jahren), häufiger zu infektiösen Komplikationen und schlechteren Outcomes führt. Ziel war es daher, Parameter zu identifizieren, welche sich prädiktiv für komplikationsreiche Verläufe zeigen.

## Studiendesign und Untersuchungsmethoden

### Studiendesign

**Figure 1:**
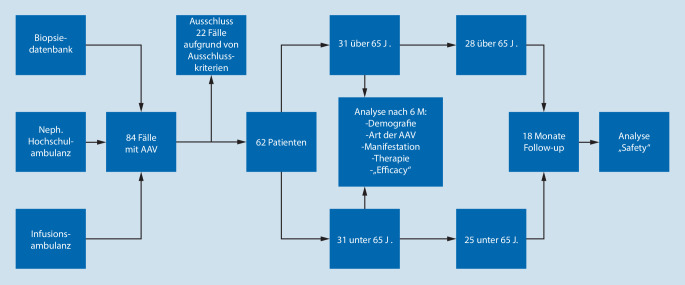

Monozentrische, retrospektive Beobachtungsstudie am Universitätsklinikum Ulm. Ausschlusskriterien waren Diagnosestellung vor 2004 und Follow-up von weniger als 6 Monaten (Abb. 1). Bei 62 identifizierten Patienten mit renaler Beteiligung wurden Daten über Therapie, Therapieansprechen (6 Monate nach Therapiebeginn), Nebenwirkungen und therapieassoziierte Komplikationen erhoben. Als Verbesserung der Nierenfunktion wurden ein Anstieg der eGFR um 5 ml/min sowie ein erfolgreiches Absetzen der Dialyse gewertet [6]. Der Birmingham Vasculitis Activity Score (BVAS) konnte bei unvollständiger Dokumentation nicht zur Beurteilung des Therapieansprechens herangezogen werden. Die Therapiesicherheit innerhalb 18 Monaten nach Therapiebeginn wurde anhand der „National Cancer Institute’s Common Terminology Criteria“ beurteilt [7]. Als SAE wurden alle Ereignisse gewertet, welche Grad III und höher waren. Bei allen Patienten > 65 Jahren wurde der Frailty-Status anhand von 23 standardmäßig erfassten Laborparametern, darunter u. a. Nierenfunktion, Blutbild, CRP, Albumin und Proteinurie, retrospektiv erhoben [8].

### Statistische Methoden

Für kontinuierliche Variablen wurden Mittelwerte und Standardabweichung ermittelt. Der Vergleich, der als normalverteilt ermittelten Variablen, erfolgte mittels *t*-Test für unverbundene Stichproben. Für nichtnormalverteilte Variablen erfolgte der Vergleich mittels Mann-Whitney‑U Test. Für kategorische Variablen wurden die Häufigkeiten angegeben und deren prozentualer Anteil berechnet. Der Vergleich kategorischer Variablen erfolgte mittels Chi-Quadrat-Test nach Pearson bzw. mittels Exakten Test nach Fisher. Als statistisch signifikant wurden *p*-Werte ≤ 0,05 angesehen. Für univariate und multiple logistische Regressionen zur Ermittlung von Prädiktoren wurden die „odds ratio“ (OR) sowie das 95 %-Konfidenzintervall (KI) angegeben. Die Durchführung der multiplen logistischen Regression erfolgte, nachdem relevante Korrelation und Multikollinearität der einzelnen Variablen ausgeschlossen wurde.

## Ergebnisse

### Charakteristika und Demografie

Analog zu anderen epidemiologischen Studien waren die Nachweise von *p*-ANCA (Myeloperoxidase, MPO) bei älteren Patienten (64,5 %) und der Nachweis von c‑ANCA (Proteinase 3, PR3) bei jüngeren Patienten (64,5 %) häufiger [9]. Im Vergleich zu älteren Patienten bestand bei jüngeren Patienten zum Zeitpunkt der Diagnosestellung eine signifikant bessere Nierenfunktion. Arthralgien und Myalgien wurden häufiger von jüngeren Patienten berichtet. Bezüglich der übrigen Organmanifestationen zeigten sich keine signifikanten Unterschiede zwischen beiden Gruppen (Tab. 1).

**Table 1:** 

Parameter	< 65 Jahre	> 65 Jahre	Gesamt	*p*-Wert
	31 (50 %)	31 (50 %)	62 (100 %)	
Männlich, *n* (%)	20 (64,5)	18 (58,1)	38 (61,3)	0,60
Alter bei Diagnose	51,5 ± 10,7	73 ± 4,6	62,3 ± 13,6	*<* *0,01*
c‑ANCA (PR3) (%)	20 (64,5)	9 (29,0)	29	*<* *0,01*
p‑ANCA (MPO) (%)	11 (35,6)	20 (64,5)	31 (50)	*0,02*
ANCA, negativ (%)	0 (0)	2 (6,5)	2 (3,2)	0,25
eGFR [ml/min]	30,8 ± 22,6	16,5 ± 11,5	23,7 ± 19,2	*<* *0,01*
Dialysepflichtig (%)	9 (29)	5 (16,1)	14 (22,6)	0,22
Proteinurie (%)	30 (96,8)	28 (100)	58 (98,3)	0,34
Pulmonale Beteiligung (%)	11 (35,5)	17 (54,8)	28 (45,2)	0,13
Myalgie (%)	14 (45,2)	5 (16,1)	19 (30,6)	*0,01*
Arthralgie (%)	16 (51,6)	5 (16,1)	21 (33,9)	*<* *0,01*
Fieber (%)	8 (25,8)	7 (22,6)	15 (24,2)	0,77
Fatigue (%)	20 (64,5)	18 (58,1)	38 (61,3)	0,60
Hautbeteiligung (%)	6 (19,4)	7 (22,6)	13 (21,0)	0,76
Beteiligung, Auge (%)	5 (16,1)	1 (3,2)	6 (9,7)	0,09
Beteiligung, oberer Respirationstrakt (%)	12 (38,7)	11 (35,5)	23 (37,1)	0,79
Beteiligung, Gehör (%)	5 (16,7)	4 (12,9)	9 (14,8)	0,68
Neurologische Beteiligung (%)	3 (10)	5 (16,1)	8 (13,1)	0,48
Anämie (%)	23 (79,3)	31 (100)	54 (90)	*<* *0,01*

*eGFR* glomeruläre Filtrationsrate (nach CKD-EPI-GFR-Formel), *ANCA* Antineutrophile cytoplasmatische Antikörper

### Art und Dauer der Therapie

Für die Induktionstherapie wurde am häufigsten Cyclophosphamid verwendet (72,6 %). Die Anzahl der Patienten, welche eine Therapie mit Cyclophosphamid oder Rituximab erhielten, unterschied sich nicht zwischen den beiden Altersgruppen. Hinsichtlich der Dauer einer Glukokortikoiddosis über 5 mg/Tag ergaben sich keine signifikanten Unterschiede zwischen den Altersgruppen (Tab. 2). Die Erhaltungstherapie erfolgte in beiden Altersgruppen vorwiegend mit Azathioprin. Bei älteren Patienten kam es häufiger zu einem Abbruch der Erhaltungstherapie mit Azathioprin, auch wenn dies statistisch nicht signifikant war (Tab. 2).

**Table 2:** 

Parameter	< 65 Jahre	> 65 Jahre	Gesamt	*p*-Wert
Induktion mit CYC (%)	23 (74,2)	22 (71,0)	45 (72,6)	0,78
Gesamtdosis, CYC [mg/kgKG]	121 ± 42,6	126 ± 82,1	123 ± 65,2	0,78
Induktion mit RTX (%)	9 (29)	11 (35,5)	20 (32,3)	0,59
Gesamtdosis RTX [g]	2,33 ± 0,5	2,3 ± 0,5	2,3 ± 0,5	0,89
Plasmaaustausch (%)	9 (30,0)	6 (19,4)	15 (24,6)	0,33
Plasmaaustausch (Anzahl)	6,33 ± 3,4	5,33 ± 1,9	5,93 ± 2,8	0,52
Dauer, Cortison > 5 mg/Tag [Tage]	152 ± 74	122 ± 60	137 ± 67	0,13
AZA, Erhaltung (%)	26 (86,7)	28 (96,6)	54 (91,5)	0,17
RTX, Erhaltung (%)	4 (13,3)	1 (3,4)	5 (8,5)	0,17
MMF, Erhaltung (%)	2 (6,7)	6 (20,7)	8 (13,6)	0,12

*CYC* Cyclophosphamid, *RTX* Rituximab, *AZA* Azathioprin, *MMF* Mycophenolatmofetil

### Therapieansprechen

Das Therapieansprechen wurde nach 6 Monaten bewertet. Die absolute eGFR nach abgeschlossener Induktion zeigte bei den jüngeren Patienten eine bessere Nierenfunktion. Die Veränderung der GFR (∆ GFR) unterschied sich jedoch nicht. Bei der Entwicklung eines negativen ANCA-Titers bestand zwischen den Gruppen kein signifikanter Unterschied (Tab. 3). Bei 23 % der Patienten bestand die Notwendigkeit eines Therapieabbruches oder einer Therapiepause. Bei 8 % wurde die Therapie geändert. Es ergaben sich keine signifikanten Unterschiede zwischen den Altersgruppen (Tab. 3). Gründe für einen Abbruch bzw. eine Umstellung der Induktionstherapie waren in erster Linie das Auftreten von SAE, in zweiter Linie ein fehlendes Ansprechen auf die Therapie (Daten nicht gezeigt).

**Table 3:** 

Parameter	< 65 Jahre	> 65 Jahre	Gesamt	*p*-Wert
Rückläufige ANCA (%)	28 (96,6)	29 (100)	57 (98,3)	0,31
ANCA, negativ (%)	9 (31,0)	15 (51,7)	24 (41,4)	0,11
Verbesserte Nierenfunktion (%)	24 (82,8)	25 (80,6)	49 (81,7)	0,83
GFR [ml/min]	42,3 ± 23,6	29,3 ± 15,3	35,6 ± 14,8	*0,01*
∆GFR [ml/min]	14,0 ± 17,9	12,8 ± 11,6	13,3 ± 14,8	0,75
Abbruch/Unterbrechung, Induktion (%)	7 (24,1)	7 (22,6)	14 (23,3)	0,89
Umstellung, Induktion (%)	4 (13,3)	1 (3,2)	5 (8,2)	0,15
Abbruch, AZA-Erhaltung (%)	6 (20,7)	9 (31,0)	15 (25,9)	0,37

*ANCA* antinukleäre zytoplasmatische Antikörper, *GFR* glomeruläre Filtrationsrate, *AZA* Azathioprin, *ANCA* Antineutrophile cytoplasmatische Antikörper

### Therapiesicherheit

Insgesamt traten 122 Komplikationen (AE) bei 53 Patienten auf, davon wurden 42 als schwere Komplikationen (SAE) eingeordnet. Die häufigsten Komplikationen waren Infektionen, darunter am häufigsten Pneumonien (52 %). 93,5 % der Patienten erhielten eine Cotrim (Co-trimoxazol)-Prophylaxe und 96,7 % Protonenpumpeninhibitoren, ohne Unterschied zwischen den beiden Altersgruppen. Das Auftreten von SAE war unabhängig von Alter, Geschlecht und Vaskulitisform (Daten nicht gezeigt).

Die Dauer einer Glukokortikoiddosis von mehr als 5 mg/Tag war bei Patienten mit SAE tendenziell höher (155 ± 67 vs. 120 ± 72 Tage). Patienten mit SAE waren seltener ANCA-negativ als Patienten ohne SAE (22,7 % vs. 44,8 %; *p* = 0,1).

Die Komplikationsrate von Patienten > 65 Jahren, die als frail bzw. nicht-frail eingestuft wurden, unterschied sich nicht signifikant (0,52 vs. 0,50; *p* = 0,73).

Risikofaktoren für das Auftreten einer SAE sind in Tab. 4 aufgeführt. Bei Patienten mit SAE lag signifikant häufiger eine pulmonale Beteiligung vor. Auch die Abbruchrate von Cyclophosphamid war bei Patienten mit SAE signifikant höher (39,1 % vs. 10,3 %). Patienten mit SAE hatten eine deutlich schlechtere Nierenfunktion (eGFR 28,6 ± 17,6 ml/min vs. 41,6 ± 23,1 ml/min; *p* = 0,03) und eine geringere Verbesserung der Nierenfunktion (6,4 ± 13,9 ml/min vs. 18,3 ± 14,9 ml/min; *p* < 0,01).

**Table 4:** 

Parameter	Kein SAE	Mit SAE	Gesamt	*p*-Wert
Anzahl, *N*	30	23	53	–
Pulmonale Beteiligung (%)	8 (26,7)	14 (60,9)	22 (41,5)	*0,01*
Gesamtdosis, CYC [mg/kgKG]	163 ± 73	90 ± 29	127 ± 67	*<* *0,01*
Abbruch/Unterbrechung, Induktion (%)	3 (10,3)	9 (39,1)	12 (23,1)	*<* *0,01*
eGFR [ml/min]	41,6 ± 23,1	28,6 ± 17,6	36 ± 21,7	*0,03*
∆eGFR [ml/min]	18,3 ± 14,9	6,4 ± 13,9	13,1 ± 15,5	*<* *0,01*

*SAE* „severe adverse event“, *CYC* Cyclophosphamid, *eGFR* glomeruläre Filtrationsrate (nach CKD-EPI-GFR-Formel)

Mittels univariater und multipler logistischer Regression wurden mögliche Prädiktoren für das Auftreten von SAE ermittelt (Tab. 5). Die Parameter pulmonale Beteiligung zum Zeitpunkt der Diagnosestellung, eGFR nach 6 Monaten sowie die Änderung der Nierenfunktion (∆GFR) nach 6 Monaten wurden in die univariate Regression aufgenommen.

**Table 5:** 

Parameter	OR univariat	*p*-Wert	OR multivariat	*p*-Wert
Pulmonale Beteiligung	4,23; KI = 1,3–13,7	*0,01*	6,9; KI = 1,7–27,8	*<* *0,01*
GFR nach Induktion	0,97; KI = 0,94–0,99	*0,04*	–	–
GFR-Verbesserung nach Induktion	0,94; KI = 0,9–0,99	*0,01*	0,93; KI = 0,88–0,98	*<* *0,01*

*OR* „odds ratio“, *KI* Konfidenzintervall, *eGFR* glomeruläre Filtrationsrate (nach CKD-EPI-GFR Formel)

Die Parameter „Abbruch oder Unterbrechung, Induktion“ und „Gesamtdosis, CYC“ wurden nicht aufgenommen, da das Auftreten eines SAE selbst zumeist Ursache ebenjener Therapieunterbrechung bzw. ebenjenes Therapieabbruches war. In der univariaten logistischen Regression waren eine pulmonale Beteiligung bei Krankheitsbeginn (OR = 4,23; KI = 1,3–13,7; *p* = 0,01), eine geringere ∆GFR (OR = 0,94; KI = 0,9–0,99; *p* = 0,01) und eine absolut geringere eGFR (OR = 0,97; KI = 0,94–0,99; *p* = 0,04) signifikante Prädiktoren für das Auftreten eines SAE. In der multiplen logistischen Regression verblieben die pulmonale Beteiligung (OR = 6,9; KI = 1,7–27,8; *p* = 0,006) und die ∆GFR (OR = 0,93; KI = 0,88–0,98; *p* = 0,005; Tab. 5).

Bei 14 Patienten traten im Verlauf 2 oder mehr SAE auf. Auch diese Patienten zeigten häufiger eine pulmonale (28,2 % vs. 78,6 %; *p* = 0,001) sowie eine neurologische Manifestation (5,3 % vs. 35,7 %; *p* < 0,01) bei Krankheitsbeginn. Sie waren häufig über 65 Jahre alt (43,6 % vs. 78,6 %; *p* = 0,025) und bekamen im Mittel länger eine Glukokortikoiddosis > 5 mg/Tag (120 ± 70 vs. 171 ± 65; *p* = 0,03). Zusätzlich bestanden bei diesen Patienten eine geringere eGFR [ml/min] (39,5 ± 22,7 vs. 26,1 ± 15,4; *p* = 0,05) sowie ∆GFR [ml/min] nach Induktionstherapie (16 ± 14,8 vs. 4,9 ± 15; *p* = 0,02). Auch bei dieser Auswertung ergab die Betrachtung der Frailty in der Patientengruppe > 65 Jahren keinen signifikanten Unterschied (*p* = 0,81).

In der univariaten logistischen Regression konnten als Prädiktoren für das Auftreten ≥ 2 SAE eine pulmonale Beteiligung (OR = 9,33; KI = 2,18–39,9; *p* < 0,01), Alter über 65 Jahre bei Diagnosestellung (OR = 4,75; KI = 1,14–19,7; *p* = 0,03) und eine längere Dauer einer Glukokortikoiddosis > 5 mg/Tag (OR = 1,011; KI = 1–1,02; *p* = 0,05) identifiziert werden. Eine Verbesserung der Nierenfunktion (anhand ∆GFR) konnte dagegen als protektiv identifiziert werden (OR = 0,95; KI = 0,9–0,99; *p* = 0,03).

## Diskussion

In dieser monozentrischen retrospektiven Studie wurden bei Patienten mit ANCA-assoziierter Vaskulitis, abhängig vom Lebensalter, Therapieansprechen und Sicherheit der immunsuppressiven Therapie ausgewertet. Unsere wichtigsten Ergebnisse zeigten, dass ältere (> 65 Jahre) und jüngere (< 65 Jahre) Patienten gleich gut auf die Therapie ansprachen. Wir zeigten, dass SAE insgesamt zwar unabhängig vom Alter der Patienten auftraten, ältere Patienten jedoch signifikant häufiger ≥ 2 SAE entwickelten, wobei Pneumonien die häufigsten infektiösen SAE darstellten. Dies bestätigt unsere Hypothese, dass ältere Patienten eher zu komplikationsreichen Verläufen neigen und deckt sich mit Ergebnissen anderer Studien, die insbesondere eine deutlich erhöhte Rate an infektiösen Komplikationen bei älteren Patienten zeigen konnten [10]. Wichtiger Risikofaktor für das Auftreten einer oder mehrerer SAE war eine pulmonale Beteiligung. Dieser Zusammenhang wurde auch in anderen Studien beschrieben [11–13]. Erklärungen hierfür könnten eine intensivere Immunsuppression in dieser Gruppe sowie eine erhöhte Anfälligkeit der geschädigten Lunge gegenüber Infekten sein [12, 13]. Darüber hinaus war die Dauer einer Glukokortikoiddosis > 5 mg/Tag ein Risikofaktor für das Auftreten von 2 oder mehr SAE. Eine Verbesserung der Nierenfunktion nach der Therapie zeigte sich hingegen protektiv.

Unsere Ergebnisse bestätigen damit andere Studien, welche v. a. die Dauer der Glukokortikoidtherapie sowie die Entwicklung der GFR als Risikofaktoren für infektiöse Komplikationen identifizieren konnten [14–16]. Dies ist insofern interessant, da die Dauer der Glukokortikoidtherapie in unserer Analyse und in anderen Studien keinen signifikanten Einfluss auf Remissionserhalt, Rezidive und die Entwicklung einer terminalen Niereninsuffizienz hatte [17]. Dies stimmt mit den neuesten Ergebnissen der PEXIVAS-Studie überein, welche keine Vorteile in einer prolongierten Glukokortikoidtherapie zeigte, bei gleichzeitig erhöhter Rate an infektiösen Komplikationen [18]. Neue Medikamente wie der C5a-Rezeptor-Inhibitor Avacopan könnten das Potential haben Glukokortikoide bei Patienten mit AAV schneller auszuschleichen bzw. zu ersetzen, wobei langfristige Daten zur Sicherheit noch fehlen [19].

Das Alter der Patienten hatte keinen Einfluss auf die Wahl der Induktions- oder Erhaltungstherapie. Beide Gruppen erhielten vornehmlich Cyclophosphamid als Induktionstherapie. Auch hinsichtlich der Therapiedauer sowie der Dauer einer Glukokortikoid-Dosis über 5 mg/Tag, ergaben sich keine signifikanten Unterschiede. Dies bestätigt die im klinischen Alltag gemachte Erfahrung, dass ältere Patienten bei leitliniengerechter Behandlung keine veränderte Therapie erhalten [20].

Andere Studien, die den Verlauf von AAV abhängig vom Alter untersucht haben, zeigten eine deutlich erhöhte Mortalität bei älteren Patienten [9, 21]. Aufgrund des Studiendesigns wurde die Mortalität in unserer Studie nicht erfasst.

Unsere Analyse der Frailty konnte keine signifikanten Unterschiede hinsichtlich des Therapieansprechens und des Auftretens von SAE in der Patientengruppe > 65 Jahren zeigen. Einschränkend sind hier die mit 31 Patienten niedrige Fallzahl der > 65-Jährigen sowie die Verwendung eines laborbasierten Frailty-Index anzumerken. Letzterer zeigte sich bei Beurteilung des Entlasslabors besser prädiktiv für die Einjahresmortalität als bei Beurteilung des in unserer Arbeit verwendeten Aufnahmelabors [22]. Aktuelle Daten zeigen durchaus einen Zusammenhang von Frailty mit komplikationsreichen Verläufen bei AAV [23]. Dies deckt sich mit Ergebnissen, die einen negativen Einfluss von Frailty auf die Prognose von z. B. nephrologischen oder chirurgischen Patienten zeigen [24, 25]. Umgekehrt scheint eine chronische Inflammation die Entwicklung von Frailty zu begünstigen [26, 27]. Rheumatologische Erkrankungen sind somit auch wesentlich an der Pathogenese von Frailty beteiligt [28]. Eine Beurteilung des Frailty-Status könnte daher auch bei jüngeren Patienten mit AAV zur Risikostratifizierung beitragen. Weitere Studien sind hier jedoch notwendig.

Limitationen unserer Studie sind das retrospektive Design sowie die Art der Patientenidentifikation. Dadurch bestand ein möglicher „survivorship bias“. Patienten, welche kurz nach Diagnose während der initialen stationären Behandlung verstarben (die Frühmortalität beträgt ca. 10 % [29]), wurden somit nicht erfasst. Diese erfüllten, bei einem geplanten Follow-up von mindestens 6 bzw. 18 Monaten, die Einschlusskriterien der Studie ohnehin nicht.

Um die Qualität unserer retrospektiv gesammelten Daten zu validieren, wurden diese mit den Daten prospektiv durchgeführter Studien hinsichtlich der Anzahl aufgetretener SAE verglichen. Hierbei zeigten sich zwischen unserer Kohorte und den Kohorten der verglichenen Studien keine relevanten Unterschiede hinsichtlich des Auftretens von AE und SAE (Zusatzmaterial online: Tab. 1).

## Fazit für die Praxis

- Ältere und jüngere Patienten unterscheiden sich hinsichtlich der Therapie der AAV nicht. Bei älteren Patienten treten jedoch häufiger mehrere SAE auf. Wir fanden eine starke Korrelation zwischen pulmonaler Beteiligung und einem komplizierten Verlauf. Außerdem konnte eine längere Glukokortikoidtherapie als Risikofaktor für das Auftreten von SAE identifiziert werden.
- Prospektive kontrollierte randomisierte Studien mit Augenmerk auf ältere Patienten sollten initiiert werden, um neue Therapiekonzepte zu erarbeiten. Hier zu nennen ist beispielsweise Avacopan, ein C5a-Rezeptor-Inhibitor, der das Potenzial hat, Glukokortikoide bei Patienten mit AAV schneller auszuschleichen bzw. zu ersetzen. Daten zur langfristigen Sicherheit fehlen jedoch noch.

### Supplementary Information




