# Supplementary material for: Treatment response and complications of older patients with ANCA(antineutrophil cytoplasmatic antibody)-associated vasculitis
Source: Z Gerontol Geriatr. 2022 Dec 19;56(8):661–6. [Article in German] doi: 10.1007/s00391-022-02145-0 (PMC10709238; doi:10.1007/s00391-022-02145-0)
Supplement: Supplementary file 1 [file 391_2022_2145_MOESM1_ESM.docx]

**Supplementary Tabelle 1:** Rate von Adverse Event oder Severe Adverse Event in den Studienkohorten RAVE, CYCLOPS, RITUXVAS Studie und Ulmer Kohorte

| Komplikation | RAVE | CYCLOPS | RITUXVAS | Uni Ulm |
| --- | --- | --- | --- | --- |
| AE | 99 % | 77 % | N.A. | 85 % |
| SAE | 40 % | 35% | 39 % | 43% |

*AE: Adverse event; SAE: Severe adverse event.*
